# Granzyme B PET Imaging in Response to In Situ Vaccine Therapy Combined with αPD1 in a Murine Colon Cancer Model

**DOI:** 10.3390/pharmaceutics14010150

**Published:** 2022-01-08

**Authors:** Siddesh V. Hartimath, Boominathan Ramasamy, Tan Yun Xuan, Tang Jun Rong, Shivashankar Khanapur, Peter Cheng, You Yi Hwang, Edward G. Robins, Julian L. Goggi

**Affiliations:** 1 Laboratory of Radiochemistry & Molecular Imaging (LRMI), Institute of Bioengineering and Bioimaging (IBB), A*STAR Research Entities, Helios, Singapore 138667, Singapore; Boominathan_Ramasamy@ibb.a-star.edu.sg (B.R.); Tan_Yun_Xuan@ibb.a-star.edu.sg (T.Y.X.); Tang_Jun_Rong@ibb.a-star.edu.sg (T.J.R.); Shivashankar@ibb.a-star.edu.sg (S.K.); Peter_Cheng@ibb.a-star.edu.sg (P.C.); edward_robins@ibb.a-star.edu.sg (E.G.R.); 2 Department of Pharmacology, Faculty of Pharmaceutical Sciences, UCSI University, Kuala Lumpur 56000, Malaysia; 3 FACS facility, Singapore Immunology Network (SIgN), A*STAR Research Entities, Immunos, Singapore 138665, Singapore; leon_hwang@immunol.a-star.edu.sg; 4 Clinical Imaging Research Centre (CIRC), Yong Loo Lin School of Medicine, National University of Singapore, Singapore 117599, Singapore

**Keywords:** granzyme B, PET imaging, immunotherapy, CpG-ODN, [^18^F]AlF-mNOTA-GZP

## Abstract

Immune checkpoint inhibitors (ICIs) block checkpoint receptors that tumours use for immune evasion, allowing immune cells to target and destroy cancer cells. Despite rapid advancements in immunotherapy, durable response rates to ICIs remains low. To address this, combination clinical trials are underway assessing whether adjuvants can enhance responsiveness by increasing tumour immunogenicity. CpG-oligodeoxynucleotides (CpG-ODN) are synthetic DNA fragments containing an unmethylated cysteine-guanosine motif that stimulate the innate and adaptive immune systems by engaging Toll-like receptor 9 (TLR9) present on the plasmacytoid dendritic cells (pDCs) and B cells. Here, we have assessed the ability of AlF-mNOTA-GZP, a peptide tracer targeting granzyme B, to serve as a PET imaging biomarker in response to CpG-ODN 1585 in situ vaccine therapy delivered intratumourally (IT) or intraperitoneally (IP) either as monotherapy or in combination with αPD1. [^18^F]AlF-mNOTA-GZP was able to differentiate treatment responders from non-responders based on tumour uptake. Furthermore, [^18^F]AlF-mNOTA-GZP showed positive associations with changes in tumour-associated lymphocytes expressing GZB, namely GZB+ CD8+ T cells, and decreases in suppressive F4/80+ cells. [^18^F]AlF-mNOTA-GZP tumour uptake was mediated by GZB expressing CD8+ cells and successfully stratifies therapy responders from non-responders, potentially acting as a non-invasive biomarker for ICIs and combination therapy evaluation in a clinical setting.

## 1. Introduction

Immunotherapy has emerged as the fourth pillar of cancer treatment, along with chemotherapy, radiotherapy, and surgery. Immunotherapy exploits the host immune system, activating it to identify and destroy cancer cells [1]. Immune checkpoint inhibitors (ICIs) block the checkpoint receptors that tumours use to evade the immune system, dampening T cell activation. However, despite rapid advancements in immunotherapy, durable response rates to ICIs remain low, especially in colorectal cancers which are typically microsatellite stable [2]. In order to address this, combination clinical trials are ongoing to assess which adjuvants can enhance responsiveness by increasing tumour immunogenicity.

CpG-oligodeoxynucleotides (CpG-ODN) are small synthetic DNA fragments containing unmethylated cysteine-guanosine motif dinucleotides that stimulate the innate and adaptive immune systems. These CpG-ODNs can mimic the immunostimulatory activity of bacterial DNA, activating Toll-like receptor 9 (TLR9) [3,4,5,6] present on the endosomes of plasmacytoid dendritic cells (pDCs) and B cells [5]. This activation induces a range of responses, including IFN-α release from pDCs, IFN-γ from NK cells, and the promotion of the T helper (Th)1 response [7]. Additionally, CpG-ODN can stimulate the production of interleukin-6 (IL6) and the proliferation of B cells, thereby upregulating co-stimulatory molecules on antigen-presenting cells (APCs) and stimulating myeloid lineage cells, such as macrophages, monocytes, and conventional dendritic cells [DCs] [8]. Because of these properties, CpG-ODN has been explored as an adjuvant for immune modulators to treat solid cancers [9,10]. Profound anti-tumoural activity has been observed when CpG-ODN is administered locally into the tumour [9] and enhanced when combined with either immunotherapy or radiotherapy [10,11], leading to their clinical assessment as in situ vaccines [11,12,13,14,15,16].

Non-invasive assessment of response to these novel combination therapies is complicated. Currently, there are a lack of specific biomarkers capable of providing a readout of in situ immune responses to different treatment strategies, complicating interpretation of clinical trials combining different treatment strategies, such as in situ vaccines and ICIs. Numerous studies have demonstrated that tumour-associated immune infiltrates are required for immune therapy efficacy, and numerous imaging biomarkers have been developed to quantify immune cell infiltrates, such as the T lymphocyte populations CD3 and CD8. However, these biomarkers provide little context and can be negatively affected by immune suppression. Granzyme B is a serine protease released by active NK cells and CD8+ cytotoxic T-cells along with the pore-forming protein perforin to induce apoptosis in tumours providing information on tumouricidal activity. We have previously characterised a PET imaging-based peptide biomarker targeting granzyme B ([^18^F]AlF-mNOTA-GZP) and demonstrated that it can successfully stratify tumours responding to combinations of immune therapeutics from non-responding tumours [17,18]. In the current study, we have evaluated whether [^18^F]AlF-mNOTA-GZP, can also be used for the stratification of response to CpG-ODN in situ vaccine therapy combined with the immune checkpoint inhibitor αPD1 in a syngeneic mouse model of colon cancer. Furthermore, we explored whether different routes of administration of CpG-ODN could influence different tumour-associated immune responses, correlating tumour uptake of [^18^F]AlF-mNOTA-GZP to differences in tumour-infiltrating immune cells using FACS.

## 2. Materials and Methods

### 2.1. General Information

All reagents were purchased from commercial suppliers and used without further purification. H-Asp (OtBu)-H NovaSyn TG resin (0.21 mmol/g) was obtained from Merck (Singapore). Fmoc-amino acids, HATU and HOAt, were obtained from Advanced Chemtech (Sabah, Malaysia). Fmoc-glutamic acid was t-butyl protected. (p-SCN-Bn)-NOTA was purchased from Macrocyclics (Plano, TX, USA). Sep-Pak^®^ light (46 mg) Accell™ plus QMA carbonate cartridges and Sep-Pak^®^ C18 light cartridges were purchased from Waters Corporation (Milford, CT, USA). Saline solution (0.9% *w*/*v*) was purchased from Braun Medical Industries (Singapore). All other chemicals and reagents were purchased from Sigma-Aldrich (Singapore), Fisher Scientific (Waltham, MA, USA) and Tokyo Chemical Industry (Chuo-ku, Tokyo, Japan). Carrier free aqueous [^18^F] fluoride was produced via the [^18^O(p,n)^18^F] nuclear reaction (GE PETtrace 860 cyclotron, Boston, MA, USA). Quality control of radiolabelled tracer was performed on a UFLC Shimadzu HPLC (Kyoto, Japan) system equipped with a dual-wavelength UV detector and a PMT-radio detector (Flow-Ram, Lab Logic, Broomhill, Sheffield, UK). Radioactivity measurements were made with a CRC-55tPET dose calibrator (Capintec, Florham Park, NJ, USA).

### 2.2. Radiochemistry of [^18^F]AlF-mNOTA-GZP

The synthesis of NOTA–β-Ala–Gly–Gly–Ile–Glu–Phe–Asp–CHO (mNOTA-GZP) was prepared using standard Fmoc chemistry characterisation was done by using HPLC and mass spectroscopy as previously described [17]. Radiochemistry of [^18^F]AlF-mNOTA-GZP was prepared as previously described [18]. [^18^F]AlF-mNOTA-GZP was prepared as a colourless solution (10% ethanol in saline, pH = 7.4) with a non-decay corrected radiochemical yield of 19–25% (reaction time ~50 min). The purity of the radiotracer was >98%, and molar activity was 45 ± 20 GBq/µmol (*n* = 6).

### 2.3. CpG-Animal Model

All animal procedures were carried out according to Institutional Animal Care and Use Committee Singapore (IACUC No. 181399) and NIH guidelines. Five- to seven-week-old female BALB/c mice were purchased from InVivos (Singapore). A CT-26 murine colon model was developed as described previously [18]. CT-26 cells (0.2 M) were implanted subcutaneously (s.c) into the right upper limb of BALB/c mice. Once the tumour had grown approximately 100 mm^3^ (after 5 days of implantation). The animals were randomised into treatment groups, treated either with rat IgG2a isotype control antibody, CpG-ODN monotherapy (300 μg of CpG-ODN 1585 oligonucleotide (Integrated DNA technology) (Leuven, Belgium) delivered either via intratumoural (IT) or intraperitoneal (IP) route on days 5, 8 and 11), αPD1 monotherapy (αPD1 mAb clone RMP1-14 (Bio-X Cell, Lebanon, NH, USA 10 mg/kg, IP) on days 6, 9 and 12), or a combination therapy groups (αPD1 & CpG-ODN (IT) or αPD1 & CpG-ODN (IP) as per the regimen described above (Supplementary Figure S1A). Animals were monitored for tumour growth using callipers on days 5, 8, 12, 15 and 20 after tumour implantation. Tumour volume was calculated using the modified ellipsoid formula 1/2(Length × Width^2^) [19]. Tumour growth inhibition (%TGI) was determined using the formula TGI (%) = (Vc − Vt)/(Vc − Vo) × 100, where Vc and Vt are the mean tumour volume of control and treated groups at the end of the study and Vo at the start.

### 2.4. Small Animal PET-CT Imaging

As previously described, small animal PET-CT imaging was performed 14 days after tumour inoculation using a Siemens Inveon PET-CT [18]. Mice were anaesthetised using isoflurane gas (maintained at 1.2% alveolar concentration) and injected with [^18^F]AlF-mNOTA-GZP (~15 MBq) via the lateral tail vein. A 10-min static PET acquisition was performed at 60 min post-injection (p.i.) of the tracer, and a CT scan was acquired for anatomical co-registration. Animals were monitored during imaging using the BioVet physiosuite for their body temperature and respiration rate. The reconstruction and the data analysis were performed as described previously [17,18] using Amide software (version 10.3 Sourceforge). The radiotracer uptake in tissues was determined and normalised to injected dose and converted to a percentage of injected dose per gram of tissue (% ID/g).

### 2.5. Fluorescence-Assisted Cell Sorting (FACS)

Tumours were excised immediately after in vivo PET imaging and freshly processed for flow cytometry. A single-cell suspension was generated by incubating in modified RPMI (Gibco) supplemented with 10% heat-inactivated fetal bovine serum (Gibco, Life Technologies, Waltham MA, USA), 20 µg/mL of DNAse1 (Sigma-Aldrich, Singapore) and 200 µg/mL of Collagenase (Sigma-Aldrich, Singapore). The samples were mechanically diced and incubated for 1 h at 37 °C and dissociated into single cells by passing through a 100-µm cell strainer. The samples were then counted and assessed for viability with Trypan Blue using hemocytometer (Sigma-Aldrich, Singapore). One million (1 M) viable cells from each sample were plated into a 96-well plate, and cells were stained for FACS analysis with different antibodies as per the manufacturer’s instructions. We looked at cell proportions as a change in percentage of either the parent population, rather than absolute cell counts. The gating strategy for the FACS analysis was presented in Supplementary Figure S2, and the gating was optimised from the spleen and tumour samples treated form the control IgG mice. The cells were stained with antibodies against CD103 (clone M290 FITC; BD Biosciences, San Jose, CA, USA), CD25 (clone PC61 BB700; BD Biosciences), CD45 (clone 30-F11 BUV395; BD Biosciences), Fixable Live/Dead Blue (Invitrogen), CD62L (clone MEL-14 BUV563; BD Biosciences), CD86 (clone GL1 BUV615; BD Biosciences), F4/80 (clone T45-2342 BUV661; BD Biosciences), NKp46 (clone 29A1.4 BUV737; BD Biosciences), CD3e (clone 500A2 BUV805; BD Biosciences), FoxP3 (clone 150D AlexaFluor647; Biolegend), CD44 (clone IM7 APC-R700; BD Biosciences), CD11b (clone M1/70 APC-Cy7; Biolegend, London, UK), granzyme B (clone QA16A02 PE; Biolegend), CCR7 (clone 4B12 PE-CF594; BD Biosciences), CD19 (clone 6D5 PE-Cy5; Biolegend), CD206 (clone C068C2 PE-Cy7; Biolegend), CD127 (clone SB/199 BV421; BD Biosciences), Ly6G (clone 1A8 BV480; BD Biosciences), CD8a (clone 53-6.7 BV510; BD Biosciences), CD11c (clone N418 BV570; Biolegend), Ly6C (clone HK1.4 BV605; Biolegend), Siglec F (clone E50-2440 BV650, BD Biosciences), CD68 (clone FA-11 BV711; Biolegend), CD4 (clone GK1.5 BV750; BD Biosciences) and I-A/I-E (clone M5/114.15.2 BV785; Biolegend).

Flow cytometry was performed on a BD FACSymphony. Fluorophore compensations and detector voltage were set up using single stains on murine spleen cells. Data was recompensated and analysed using FlowJo V10.7.1 software (FlowJo LLC, Ashland, OR, USA).

### 2.6. Statistical Analysis

All data were analysed using a 1-way ANOVA (Kruskal-Wallis) with a Dunn’s post-test using GraphPad Prism (GraphPad Software v 8.0.0, San Diego, CA, USA). Statistical significance was considered where *p* < 0.05. Data are expressed as mean ± S.D. unless otherwise indicated.

## 3. Results

### 3.1. Assessment of Treatment Efficacy Using Tumour Growth Volume

**Figure 1:**
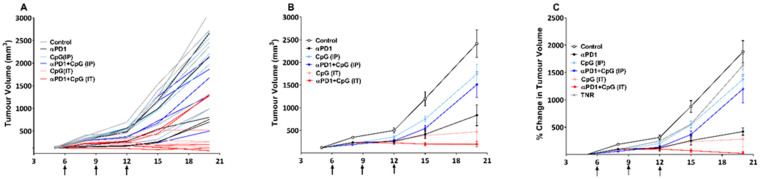
Assessment of the therapeutic effect of CpG-ODN and αPD1 combinations on changes in CT-26 tumour volume. (**A**) Individual animal tumour volume data measured on days 5, 9, 12, 15 and 20 post-implantation. (**B**) Group averaged tumour volume data from each treated cohort. (**C**) Percentage change in tumour volume after separation into TR and TNRs based on %TGI. All data are shown as mean ± S.D. (where TR, treated responder and TNR, treated non-responder). Arrow indicates the dosing of αPD-1.

Mice bearing colon CT-26 tumours were treated with control IgG, αPD1, CpG-ODN (IT), CpG-ODN (IP), or a combination of αPD1 + CpG-ODN (IP) or αPD1 + CpG-ODN (IT) (Supplementary Figure S1A) and tumour volumes measured over time. We observed that CT-26 tumour growth curves were normally distributed (Shapiro-Wilk *p* > 0.68) (Supplementary Figure S1B) and exhibited different responses to monotherapy or combination therapies based on the tumour volume measurements (individual and grouped tumour volumes are shown in Figure 1A,B). Tumour volume changes indicate that local IT CpG-ODN delivery is required for anti-tumour effects and combined αPD1 + CpG-ODN (IT) elicited even greater anti-tumour activity (Figure 1C, Supplementary Table S1).

Tumour volumes were converted to percentage tumour growth inhibition (%TGI), and [^18^F]AlF-mNOTA-GZP tumour uptake was correlated across all data sets before post hoc manipulation (Pearson r = 0.654, **** *p* < 0.0001, *n* = 60). When individual treatment groups were assessed prior to stratification, overall %TGI and tumour uptake of [^18^F]AlF-mNOTA-GZP were found to be positively correlated only in the CpG-ODN (IT)- (r = 0.703, * *p* < 0.05 *n* = 10) and αPD1 + CpG-ODN (IT)-treated groups (r = 0.806, ** *p* < 0.01, *n* = 10). The αPD-1 monotherapy cohort did not reach significant difference (r = 0.720, *p* > 0.05) due to the variability in tumour responses (<40% of response rate). Similarly, the monotherapy CpG-ODN (IP)-treated cohort (r = 0.507, *p* > 0.05) and combination therapy cohort of αPD1 + CpG-ODN (IP) (r = 0.257, *p* > 0.05) were not well correlated, perhaps due to low tumour accumulation and rapid clearance of CpG-ODN from the body (Supplementary Table S2).

An effective therapy response in this pre-clinical model was estimated by comparing the tumour volumes between day 6 (baseline) and day 20 (post-therapy volume). We separated the treatment arms into two groups based on tumour volume data, a treatment responder (where we included a partial and complete responder, TR) and a treatment non-responder (TNR). This approach has been used previously in order to assess imaging agents for their ability to stratify responders from non-responders; however, this approach may still be prone to bias [18,20,21,22,23]. Therefore, TRs were identified as final tumour volumes less than 750 mm^3^ and include tumours with stable or decreased volumes (tumour volumes are shown in Supplementary Table S1). This tumour volume was selected as this is more than 3 times the standard deviation from the mean tumour volume of the control group on day 20, and using this approach, there is only a less than 1% chance for a TR to be incorrectly assigned. Treatment response varied across the treatment arms, with αPD1 + CpG-ODN (IT) combined treated group exhibiting the most significant response rate (Supplementary Table S2).

### 3.2. Assessment of Treatment Efficacy Using [^18^F]AlF-mNOTA-GZP PET Imaging

**Figure 2:**
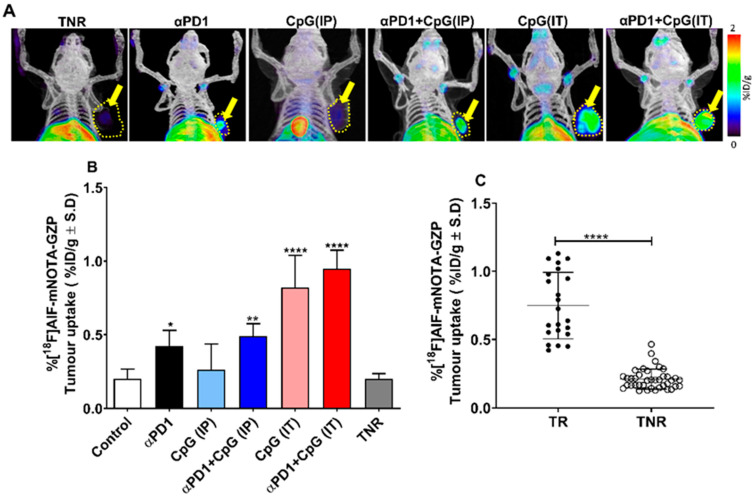
Fused PET/CT images of [^18^F]AlF-mNOTA-GZP from Balb/C mice. (**A**) MIP images showing [^18^F]AlF-mNOTA-GZP uptake in CT-26 tumours from treated non-responders (TNR), αPD1 monotherapy, CpG(IP) monotherapy, CpG(IT) monotherapy, combined αPD1 + CpG (IP) and αPD1 + CpG (IT) treated responders. Yellow arrow indicates the position of the CT-26 tumour, and dashed line indicates tumour boundary. (**B**) Graph showing [^18^F]AlF-mNOTA-GZP uptake in responders from each treatment arm compared to TNR. Significant increases in [^18^F]AlF-mNOTA-GZP tumour uptake were observed in αPD1 (* *p* < 0.05, *n* = 7) and CpG-ODN (IT) (**** *p* < 0.0001, *n* = 6) monotherapy treated arms and αPD1 + CpG-ODN (IP) (** *p* < 0.01, *n* = 5) and αPD1 + CpG-ODN (IT) (**** *p* < 0.0001, *n* = 6) combination treated arms when compared to treated non-responders (TNR, *n* = 10). No significant changes were observed in the CpG-ODN (IP) monotherapy group (*n* = 10) compared to TNRs. (**C**) Individual CT-26 tumour uptake of [^18^F]AlF-mNOTA-GZP in TR and TNRs overall (**** *p* < 0.0001, *n* = 39). Where, ● Tumour responder (TR) and o Tumour non responder (TNR). The PET imaging was done 2 days after the last PD1 administration, and all data is provided as mean ± S.D.

In-vivo PET imaging with [^18^F]AlF-mNOTA-GZP revealed that tumours could be visualised above background, and the radiotracer uptake was found to be heterogeneous across different treatment arms (Figure 2A). [^18^F]AlF-mNOTA-GZP was able to stratify responders from non-responders across different therapy arms (Table 1 and Figure 2B,C). In general, low [^18^F]AlF-mNOTA-GZP tumour uptake was observed in the control group (0.20 ± 0.06% ID/g, *n* = 10) and TNRs (0.20 ± 0.03% ID/g, *n* = 10). Significantly higher uptake was observed in all TRs from both monotherapy αPD1 (0.42 ± 0.10% ID/g, * *p* < 0.05, *n* = 7) and CpG-ODN (IT) (0.82 ± 0.21% ID/g, **** *p* < 0.0001, *n* = 6) and combination therapy αPD1 + CpG-ODN (IP) (0.49 ± 0.08% ID/g, ** *p* < 0.01, *n* = 5) and αPD1 + CpG-ODN (IT) (0.95 ± 0.12% ID/g, **** *p* < 0.0001, *n* = 6) cohorts (Figure 2B). Furthermore, no significant uptake was observed in the CpG-ODN (IP) alone group compared to the TNR group (0.26 ± 0.17 vs. 0.20 ± 0.03% ID/g, *p* = 0.779, *n* = 10).

Tracer is mostly excreted through the urinary system, and the uptake in kidneys was 1.85 ± 0.1% ID/g, with lower clearance through the hepatobiliary system (liver uptake 1.87 ± 0.34% ID/g). We observed some uptake in immune-related organs, particularly the spleen; however, no significant differences in spleen uptake were observed between responders and non-responders (0.81 ± 0.02% ID/g vs. 0.65 ± 0.06% ID/g, *p* > 0.05) (Supplementary Figures S3 and S4).

### 3.3. Immune Cell Profiling by FACS and Its Correlation to [^18^F]AlF-mNOTA-GZP Tumour Uptake

**Figure 3:**
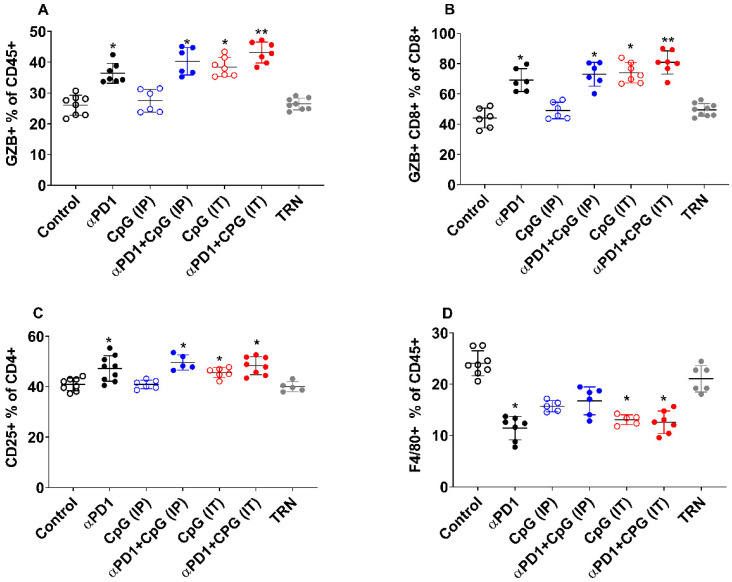
Multi-colour flow cytometry analysis of immune cells from mice bearing CT-26 tumour. Significant increases in T cell populations were observed, especially, the CD45+, CD8+ and CD4+ T cells. (**A**) Percentage of GZB+ cells relative to total CD45+ cells. A significant increase in GZB+CD45+ was noticed in all TRs from both monotherapy αPD1 (* *p* < 0.05), CpG-ODN (IT) (* *p* < 0.05), combination therapy αPD1 + CpG-ODN (IP) (* *p* < 0.05) and αPD1 + CpG-ODN (IT) (** *p* < 0.01). However, no significant increase in the GZB+CD45+T cell population was noticed in the CpG-ODN (IP) monotherapy group (*p* > 0.05). (**B**) Percentage of GZB+ CD8+ cells relative to total CD8+ cells. Similarly, a significant infiltration of GZB+CD8+ cells was observed in αPD1 (* *p* < 0.05), CpG-ODN (IT) (* *p* < 0.05) and in combination therapy αPD1 + CpG-ODN (IP) (* *p* < 0.05) and αPD1 + CpG-ODN (IT) (** *p* < 0.01). Again, no significant changes in GZB+CD8+ cell were observed in the CpG-ODN (IP) treated mice (*p* > 0.05). (**C**) CD25+CD4+ cells relative to total CD4+ cells, a significant increase in CD25+CD4+ cells in all TRs was noticed when compared to TNRs; αPD1 (* *p* < 0.05), CpG-ODN (IT) (* *p* < 0.05) and combination therapy αPD1 + CpG-ODN (IP) (* *p* < 0.05), and the αPD1 + CpG-ODN (IT) (* *p* < 0.05). CpG-ODN (IP) monotherapy group did not show any significant changes in the CD25+CD4+ cell population when compared to the TNR group (40.87 ± 1.70 vs. 39.96 ± 2.01, *p* > 0.05). (**D**) Percentage of F4/80+ relative to total CD45+ cells across different treatment arms; αPD1 monotherapy (* *p* < 0.05), CpG-ODN (IT) (* *p* < 0.05) and combination αPD1 + CpG-ODN (IT) (* *p* < 0.05). However, no such reduction was observed in the CpG-ODN (IP) (*p* > 0.990) or combination αPD1 + CpG-ODN (IP) groups (*p* > 0.05) compared to TNRs. All data are represented as individual values with mean ± S.D. (*n* = 5–10 mice/group. * *p* < 0.05; ** *p* < 0.01 compared to TNR).

FACS analysis was performed on day 14 after PET imaging in order to quantify the changes in immune infiltrates in response to therapy. Figure 3 demonstrates the immune cell composition of the CT-26 tumours and shows that overall response in this study is mainly mediated by GZB releasing T cells. A significant change was noticed in T cell populations, especially the CD45+, CD8+ and CD4+ T cells. Separating them into different treatment cohorts revealed a significant increase in GZB+CD45+ T cells in all TRs from both monotherapy αPD1 (36.40 ± 3.26, * *p* < 0.05), CpG-ODN (IT) (38.41 ± 3.11, * *p* < 0.05), combination therapy αPD1 + CpG-ODN (IP) (40.28 ± 4.47, * *p* < 0.05) and αPD1 + CpG-ODN (IT) (43.12 ± 3.48, ** *p* < 0.01). However, there was no significant increase in the GZB+CD45+T cell population in the CpG-ODN (IP) alone group when compared to TNRs (27.46 ± 3.74 vs. 26.41 ± 1.90, *p* > 0.05) (Figure 3A).

Similarly, a significant increase in the GZB+CD8+ cells was observed in monotherapy αPD1 (69.16 ± 7.55, * *p* < 0.05), CpG-ODN (IT) (73.87 ± 6.70, * *p* < 0.05) and in combination therapy αPD1 + CpG-ODN (IP) (72.99 ± 7.95, * *p* < 0.05) and αPD1 + CpG-ODN (IT) (80.83 ± 7.62, ** *p* < 0.01). Again, no significant changes were noticed in the GZB+CD8+ cells numbers in the CpG-ODN (IP)-treated mice in contrast to TNRs group (49.04 ± 5.45 vs. 49.76 ± 4.19, *p* > 0.05) (Figure 3B). In addition to CD8+ cells, we noticed a profound increase in the CD25+CD4+ cells in the monotherapy αPD1 (47.20 ± 5.05, * *p* < 0.05), CpG-ODN (IT) (45.52 ± 2.00, * *p* < 0.05) and combination therapy αPD1 + CpG-ODN (IP) (49.60 ± 2.99, * *p* < 0.05) and αPD1 + CpG-ODN (IT) (48.25 ± 3.53, * *p* < 0.0) responders. Again, the CpG-ODN (IP) monotherapy group did not show any significant changes in the CD25+CD4+ cell population when compared to the TNR group (40.87 ± 1.70 vs. 39.96 ± 2.01, *p* > 0.05) (Figure 3C).

In addition, tumour responders not only increased the infiltration of GZB+ cells but at the same time, there was a concurrent significant reduction in tumour-associated suppressive cell types. F4/80+ macrophage reductions were observed in the treatment responding groups: αPD1 monotherapy (11.44 ± 2.29, *p* * < 0.05), CpG-ODN (IT) (13.05 ± 0.977, *p* * < 0.05) and combination αPD1 +CpG-ODN (IT) (12.92 ± 1.14, *p* * < 0.05). However, no such reduction was observed in the CpG-ODN (IP) (15.69 ± 1.08, *p* > 0.990) or combination αPD1 + CpG-ODN (IP) groups (16.73 ± 2.70, *p* > 0.05) compared to TNRs (21.03 ± 2.56) (Figure 3D). Interestingly, no changes were observed in NK+ cells or GZB+ NK+ cell (Supplementary Table S3). Although there was a slight increase in the GZB+NKp46+ cells population in the TRs compared to the TNRs, this did not reach statistical significance (16.01 ± 1.91 vs. 12.83 ± 2.64, *p* > 0.05). When looking at total lymphocyte cell composition as the % GZB+CD45+, most of the cell type is associated with CD8+ and is significantly higher in TRs when compared to TNRs. We also noticed a majority of NK+ cells, CD4+ and other non-T cells at the tumour site (Supplementary Table S3).

## 4. Discussion

CpG-ODN is presented as an effective adjuvant to elicit a stronger anti-tumour response when combined with immune checkpoint inhibitors. CpG-ODN has been shown to modulate the immune system through engagement with TLR9 receptors expressed on plasmacytoid dendritic cells (pDC) or B cells [6,24,25]. The presence of unmethylated cysteine-guanosine motifs on CpG-ODN can mimic bacterial DNA and act as a pathogen-associated molecular pattern (PAMP) to trigger immune responses. To date, four classes of synthetic CpG-ODNs have been described, each with distinct structural and biological properties [26]. Type-A CpG-ODNs consist of mixtures of phosphorothioate/phosphodiester backbone along with a single motif flanked by palindromic sequences of poly G at 3′ and 5′ ends, which stimulate pDCs cells to secrete IFN-α and thereby strongly activate T cells. Type-B CpG-ODNs are encoded with multiple CpG on phosphorothioate backbone and act on pDCs to secrete TNF-α, activating B cells to produce immunoglobulin M (IgM) as a first line of response to antigen exposure. Type-C CpG-ODNs combines features of both type-A and type-B, and can activate both T cells and B cells through IFN-α and IL-6 signaling, respectively. Type-P CpG-ODNs consist of two palindromic sequences of CpG motif, and act on both T cells and B cells; however, they secrete substantially higher amounts of IFN-α through pDCs cells when compared to type-C ODNs [8,26]. In this study, we have used CpG-ODN 1585, which belongs to type-A CpG-ODNs, which stimulate and activate T cells through engaging TLR9 expressed on the pDC cells.

CpG-ODN is being explored clinically for use as a vaccine adjuvant in combination with ICIs, chemo, or radiotherapy for various cancer types (metastatic prostate cancer, melanoma, or recurrent head and neck cancer) [25,27,28,29]. However, accurate assessment of immune treatment response in a clinical setting is complex [30,31]. Typically, radiological measures of tumour volume are employed to assess response to treatment in solid tumours; however, new response types associated with immune therapies, such as pseudoprogression, hyperprogression, or a dissociative response may not be accurately interpreted with these conventional response criteria [32,33].

Non-invasive molecular imaging techniques, such as PET imaging, enable the visualisation and quantification of radiopharmaceutical uptake in the lesions, allowing for the assessment of changes in the tumour microenvironment in response to different cancer immunotherapies or combination therapies. Here, we have demonstrated the feasibility of [^18^F]AlF-mNOTA-GZP, a probe binding to granzyme B released by activated cytotoxic T cells, to act as a surrogate biomarker of response to CpG-ODN, alone or in combination with αPD1. CpG-ODN elicited a stronger anti-tumoural effect when administered intratumourally compared to intraperitoneally (Figure 1C), a result mirrored by greater tumour retention of [^18^F]AlF-mNOTA-GZP (Figure 2B). Local administration of CpG-ODN creates a stronger inflammatory response at the tumour site, activating tumour-associated pDCs, secreting higher amounts of IFNs, which in turn activate T cells, dendritic cells and NK cells. In addition, local administration of CpG-ODN can significantly increase the production of pro-inflammatory chemokines and cytokines, such as IP10, MIP1β, MCP5, MIP1, RANTES, JE, MCP5 and MIP1α, which play a role in the observed anti-tumour effects [9]. A previous study by Lou et al., demonstrated that systemic administration of CpG-ODN does produce an immune response; however, the activated immune cells fail to migrate to the tumour site [9]. Surprisingly, while the combination of αPD1 and CPG-ODN (when administered intratumorally) showed a small improvement in tumouricidal efficacy when measured tumour volume was measured, no significant potentiation of [^18^F]AlF-mNOTA-GZP tumour uptake was observed when compared to CpG-ODN when applied intratumorally alone. Despite this, [^18^F]AlF-mNOTA-GZP successfully stratified the responding tumours (TRs) from non-responders (TNRs). The increased tumour uptake of [^18^F]AlF-mNOTA-GZP in TRs was associated with a robust increase in tumour-associated activated T cells, especially GZB+ CD8+ cells, GZB+ CD45+ cells and CD25+ CD4 cells (Figure 3) are in line with previously reported data [34,35]. In tumour responders, we also found a substantial reduction in tumour-related F4/80+ macrophages, a cell type normally linked with an immune-suppressive microenvironment. However, further experiments will be required to fully understand the effects on different macrophage subpopulations. We had anticipated an increase in tumour-associated NK+ cells following intratumoural administration of CpG-ODN due to its indirect effects on these cell subtypes. However, none of the treatments induced significant changes in NK+ cell numbers.

Further work is needed to determine whether these data are clinically translatable. A simple amino acid substitution (IEFD to IEPD) confers selectivity for human granzyme B [22], potentially providing a platform for further development of granzyme B peptides as companion diagnostic able to simplify interpretation of clinical trials involving immune therapy combinations.

## 5. Conclusions

In summary, we have assessed the ability of [^18^F]AlF-mNOTA-GZP to stratify response to combined αPD1 therapy and in situ vaccination with CpG-ODN in a CT-26 murine colon cancer model. Therapy response was mediated by GZB expressing CD8+ cells and strongly correlated to [^18^F]AlF-mNOTA-GZP tumour uptake. Potentially, granzyme B imaging agents could be developed as a biomarker for adjuvant in situ vaccine therapy for use in combination with ICIs in a clinical setting.

## Figures and Tables

**Table 1:** Represents [^18^F]AlF-mNOTA-GZP quantitative PET uptake in CT-26 tumour. ROIs were defined from individual CT-26 tumour-bearing mice subjected to different treatment cohorts, and the uptake values were converted to % ID/g. All data indicated as mean ± S.D. of control groups, treatment responders (TR), and treatment non-responders (TNR) (where * *p* < 0.05; ** *p* < 0.01, **** *p* < 0.0001 comparing T.R. to TNR).

Uptake of [^18^F]AlF-mNOTA-GZP in CT-26 Tumour	
Control	0.20 ± 0.06
Treatment Responder (TR)	
αPD1	0.42 ± 0.10 *
CpG-ODN (IP)	0.26 ± 0.17
αPD1 + CpG-ODN (IP)	0.49 ± 0.08 **
CpG-ODN (IT)	0.82 ± 0.21 ****
αPD1 + CpG-ODN (IT)	0.95 ± 0.12 ****
Treatment Non-Responder (TNR)	0.20 ± 0.03

Where * *p* < 0.05; ** *p* < 0.01, **** *p* < 0.0001 comparing T.R. to TNR.

## Data Availability

The data presented in this study are available in the article as well as in Supplementary Materials.

## Supplementary Materials

The following are available online at https://www.mdpi.com/article/10.3390/pharmaceutics14010150/s1, Figure S1A. Graphical representation of initiation of the study followed by dosing, tumour measurement, PET imaging, and FACS. Balb/C mice (*n* = 10 per group) bearing CT-26 tumour were treated with control IgG, αPD1, CpG-ODN(IP), CpG-ODN(IT), or combinations of αPD1 + CpG-ODN(IP) or αPD1 + CpG-ODN (IT). B. CT-26 tumour growth curves showed normal distribution (Shapiro-Wilk p 0.683) and a different response to monotherapy or combination therapy. Figure S2. Representative flow cytometry gating strategy of the single-cell dissociated tumour cells. Gating strategy highlights the populations with significant differences mentioned in the main text (red boxes). This gating strategy shows flow plots derived from a control IgG treated mouse. Figure S3. The PET-derived biodistribution of [^18^F]AlF-mNOTA-GZP uptake in selected organs from the treatment responder (TR) and treatment non-responder (TNR) groups. The ROI was manually delineated, and the tracer uptake was extracted, adjusted to the injected dosage, and converted to a percentage injected dose per gram of tissue (percent ID/g). All data is based on the average of five animals with SEM. Figure S4. A maximum intensity projection (MIP) PET/CT fused image of [^18^F]AlF-mNOTA-GZP from Balb/C mice bearing CT-26 tumour responder, treated with αPD1 + CpG (IT). Tracer is excreted mainly through urinary routes, as a strong uptake in the bladder and kidney. Furthermore, there was strong absorption in the liver and gallbladder, indicating that the tracer is excreted via the hepatobiliary pathway. There is less uptake in the bone, indicating that the tracer is stable in vivo, and uptake in the responder tumour is much stronger than in the non-responder tumour. Where, T-Tumour, L-Liver, K-kidney, and B-bladder. Table S1: The table shows the summary of tumour volumes in controls, αPD1, CpG-ODN (IP), αPD1 + CpG (IP), CpG-ODN (IT), and αPD1 + CpG-ODN (IT.) treatment responders (TRs) and treatment non-responders (TNRs) across all therapy arms in the syngeneic CT-26 colon cancer model. Table S2: The tumour volume data were converted to the percentage of tumour growth inhibition (%TGI) by comparing the volumes between day 5 and day 20 for each treatment. Table S3. Table shows the tumour-associated immune cell populations from CT-26 tumour-bearing mice after 14 days of monotherapy αPD1, CpG-ODN (IP/IT) or combination therapies cohorts. Overall data presented in this study is mainly mediated by GZB-releasing T cells. Significant changes in T cell populations, especially, the CD45+, CD8+ and CD4+ T cells were observed. In addition, all treatment responders showed significant reductions in F4/80+ cells compared to TNRs and control. Data are shown as mean % of cells ± S.D. and represent *n* = 5–10 mice/group, * *p* < 0.05; ** *p* < 0.01, comparing TR to TNR.
