# Supplementary material for: Granzyme B PET Imaging in Response to In Situ Vaccine Therapy Combined with αPD1 in a Murine Colon Cancer Model
Source: Pharmaceutics. 2022 Jan 8;14(1):150. doi: 10.3390/pharmaceutics14010150 (PMC8779135; doi:10.3390/pharmaceutics14010150)
Supplement: Supplementary file 1 [file pharmaceutics-14-00150-s001.zip › pharmaceutics-1483623-supplementary.pdf]

pharmaceutics

Supplementary materials

Granzyme B PET Imaging in Response to In Situ Vaccine Therapy Combined with αPD1 in a Murine Colon Cancer Model

Siddesh V. Hartimath [1,*,†], Boominathan Ramasamy [1,2,†], Tan Yun Xuan [1], Tang Jun Rong [1], Shivashankar Khanapur [1], Peter Cheng [1], You Yi Hwang [3], Edward G. Robins [1,4] and Julian L. Goggi [1,*]

1 Laboratory of Radiochemistry & Molecular Imaging, (LRMI), Institute of Bioengineering and Bioimaging (IBB), A*STAR Research Entities, Helios, Singapore 138667, Singapore; Boominathan_Ramasamy@ibb.a-star.edu.sg (B.R.); Tan_Yun_Xuan@ibb.a-star.edu.sg (T.Y.X.); Tang_Jun_Rong@ibb.a-star.edu.sg (T.J.R.); Shivashankar@ibb.a-star.edu.sg (S.K.); Peter_Cheng@ibb.a-star.edu.sg (P.C.); edward_robins@ibb.a-star.edu.sg (E.G.R.)
2 Department of Pharmacology, Faculty of Pharmaceutical Sciences, UCSI University, Kuala Lumpur 56000, Malaysia
3 FACS facility, Singapore Immunology Network (SIgN), A*STAR Research Entities, Immunos, Singapore 138665, Singapore; leon_hwang@immunol.a-star.edu.sg
4 Clinical Imaging Research Centre (CIRC), Yong Loo Lin School of Medicine, National University of Singapore 117599, Singapore
* Correspondence: s_hartimath@ibb.a-star.edu.sg (S.V.H.); Julian_Goggi@ibb.a-star.edu.sg (J.L.G.)
† These authors contributed equally to this work.

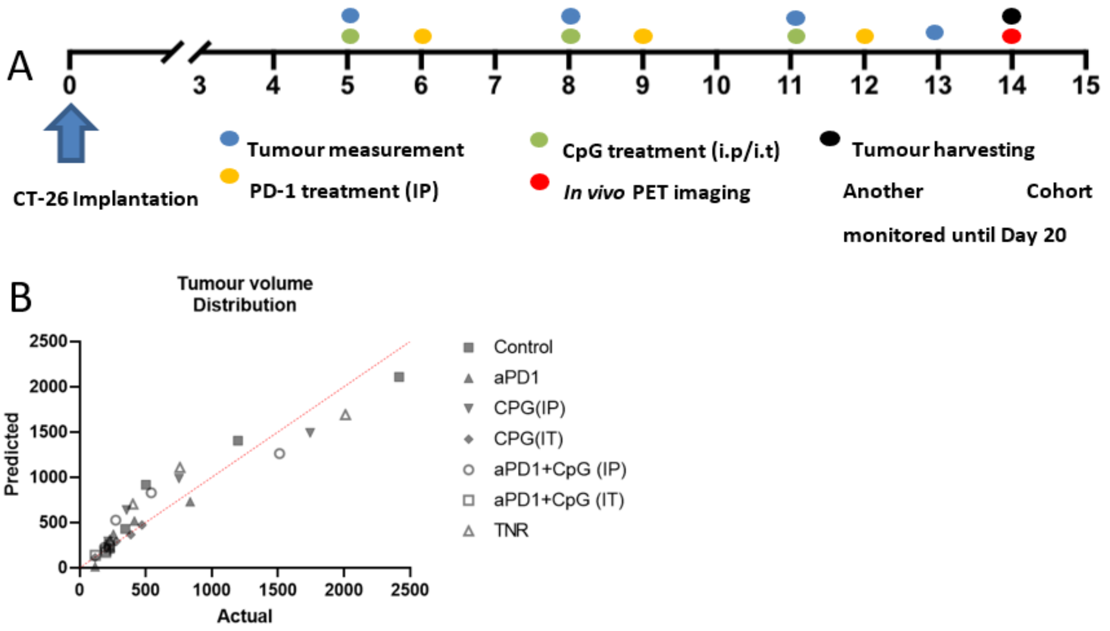

Figure S1. (**A**). Graphical representation of initiation of the study followed by dosing, tumour measurement, PET imaging and FACS. Balb/C mice (n=10 per group) bearing CT-26 tumour were treated with control IgG, αPD1, CpG-ODN(IP), CpG-ODN(IT), or combinations of αPD1 + CpG-ODN(IP) or αPD1+CpG-ODN (IT). (**B**). CT-26 tumour growth curves showing normally distributed (Shapiro-Wilk p 0.683) and showed a different response to monotherapy or combination therapy.

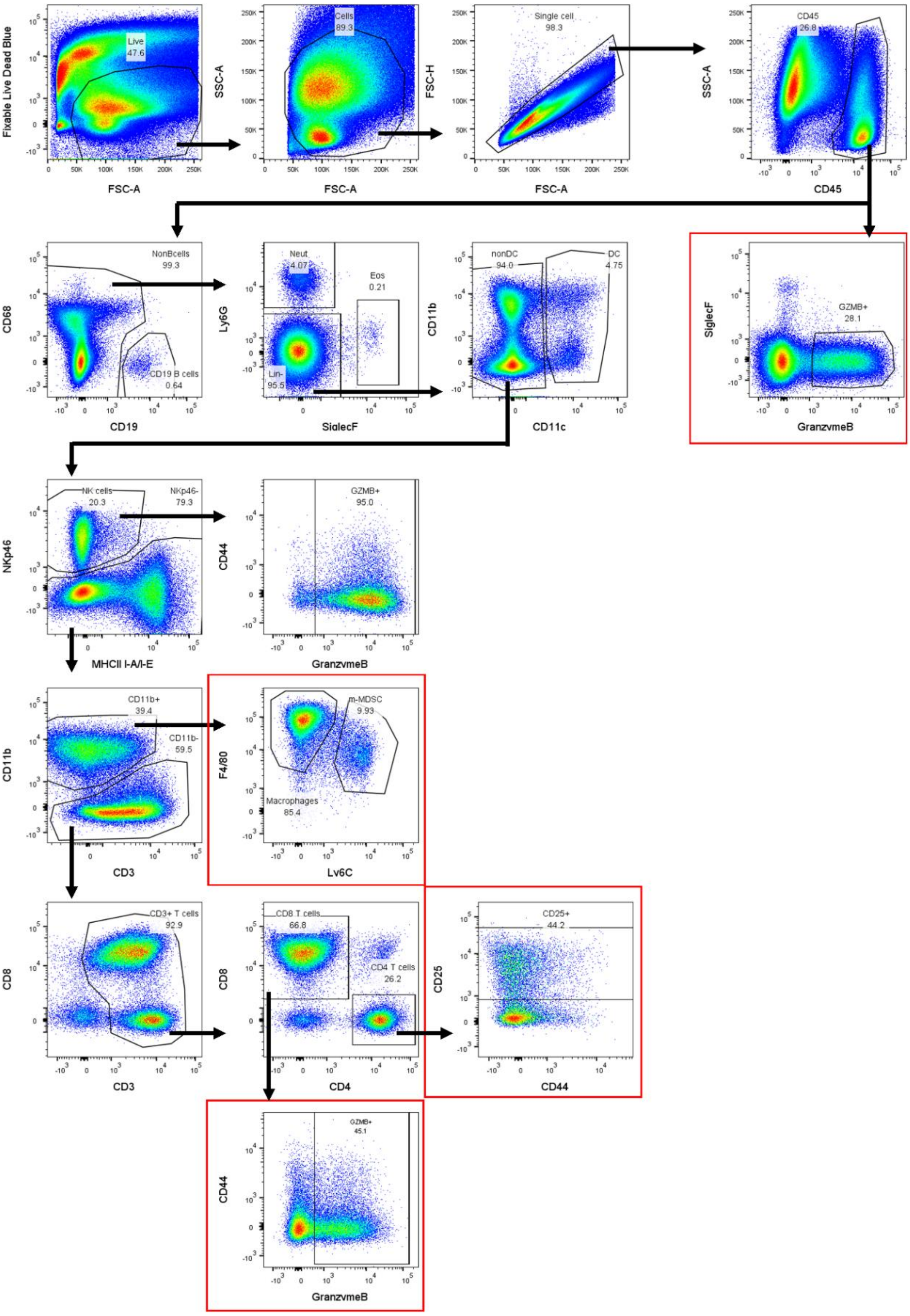

Figure S2. Representative flow cytometry gating strategy of the single cell dissociated tumour cells. Gating strategy highlights the populations with significant differences mentioned in the main text (red boxes). This gating strategy shows flow plots derived from a control IgG treated mouse.

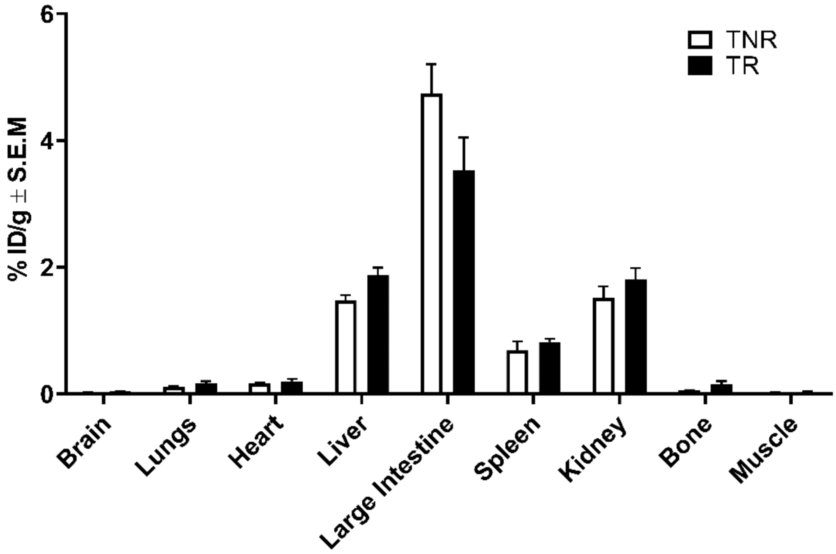

Figure S3. The PET-derived biodistribution of [^{18}F]AlF-mNOTA-GZP uptake in selected organs from treatment responder (TR) and treatment non-responders (TNR) group. The ROI was manually delineated, and the tracer uptake was extracted, adjusted to the injected dosage, and converted to a percentage injected dose per gram of tissue (percent ID/g). All data is based on the average of five animals with SEM.

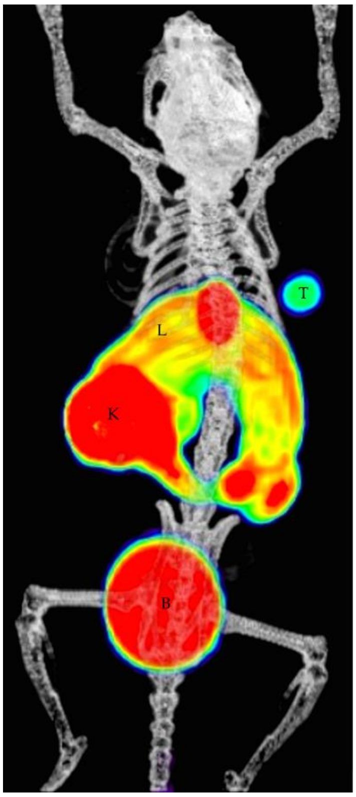

Figure S4. A maximum intensity projection (MIP) PET/CT fused image of [^{18}F]AlF-mNOTA-GZP from Balb/C mice bearing CT-26 tumour responder, treated with αPD1 + CpG (IT). Tracer is excreted mainly through urinary routes, as a strong uptake in the balder and kidney. Furthermore, there was also absorption in the liver and gallbladder, indicating that the tracer is being excreted via the hepatobiliary pathway. There is less uptake in the bone, indicating that the tracer is stable *in-vivo,* and uptake in the responder tumour is much stronger than in the non-responder tumour. Where, T—Tumour, L—Liver, K—kidney and B—bladder.

Table S1. The table shows the summary of tumour volumes in controls, αPD1, CpG-ODN (IP), αPD1+CpG (IP), CpG-ODN (IT) and αPD1 + CpG-ODN (IT.) treatment responders (TR) and treatment non-responders (TNR) across all therapy arms in the syngeneic CT-26 colon cancer model.

Treatment Cohort	Days post-inoculation	CT26 tumour volume (mm^3 ± SD)
Control	5	120.9 ± 14.0
	8	345.7 ± 48.5
	12	500.5 ± 119.7
	15	1196.8 ± 343.5
	20	2419.4 ± 677.2
Treatment Responders (TR)		
αPD1	5	116.3 ± 11.4
	8	233.3 ± 42.1
	12	255.8 ± 116.5
	15	414.6 ± 223.1
	20	834.8 ± 611.2
CpG-ODN (IP)	5	118.5 ± 13.5
	8	214.5 ± 40.9
	12	354.7 ± 85.8
	15	751.1 ± 204.5
	20	1743.5 ± 557.4
αPD1 + CpG-ODN (IP)	5	119.9 ± 14.4
	8	188.5 ± 44.1
	12	272.6 ± 97.3
	15	541.6 ± 203.7
	20	1511.6 ± 848.6
CpG-ODN (IT)	5	115.9 ± 15.5
	8	205.5 ± 69.4
	12	276.8 ± 117.2
	15	386.6 ± 188.6
	20	470.5 ± 477.8
αPD1+CpG-ODN (IT)	5	117.8 ± 14.1
	8	222.6 ± 43.9
	12	225.2 ± 106.8
	15	197.5 ± 105.1
	20	196.4 ± 166.2
Treatment Non-Responders (TNR)	5	126.8 ± 15.5
	8	274.4 ± 44.5
	12	446.5 ± 143.8
	15	897.2 ± 344.7
	20	2010.9 ± 586.1

Table S2. The tumour volume data were converted to the percentage of tumour growth inhibition (%TGI) by comparing the volumes between day 5 and day 20 for each treatment.

Treatment Arm	%TGI (mean ± SEM)
αPD1	57.02 ± 10.1
CpG(IP)	16.44 ± 6.9
αPD1+CpG(IP)	33.96 ± 11.5
CpG(IT)	74.33 ± 12.7
αPD1+CPG(IT)	90.24 ± 4.2
TNR	12.25 ± 7.9

Table S3. Table shows the tumour-associated immune cell populations from CT-26 tumour-bearing mice after 14 days of monotherapy αPD1, CpG-ODN (IP/IT) or combination therapies cohorts. Overall data presented in this study is mainly mediated by GZB releasing T cells. Significant changes in T cell populations, especially, the CD45+, CD8+ and CD4+ T cells were observed. In addition, all treatment responders showed significant reductions in F4/80+ cells compared to TNRs and control. Data are shown as mean % of cells ± S.D. and represent n = 5–10 mice/ group, * $p < 0.05$; ** $p < 0.01$, comparing TR to TNR.

	GZB+ % of CD45+	GZB+CD8+ % of CD8+	CD25+ % of CD4+	F4/80+ % of CD45	GZB+ % of NKp46+	CD3+ % of CD45+
Control	26.03 ± 3.23	44.03 ± 6.59	40.88 ± 2.51	24.04 ± 2.43	15.47 ± 2.00	41.76 ± 4.15
TR αPD1	36.27 ± 3.26*	69.16 ± 7.55*	47.21 ± 5.05*	11.44 ± 2.29*	15.34 ± 1.48	45.10 ± 6.45
CpG-ODN (IP)	27.46 ± 3.74	49.04 ± 5.45	40.84 ± 1.70	15.69 ± 1.08	16.01 ± 1.91	41.65 ± 5.75
αPD1 + CpG-ODN (IP)	40.28 ± 4.47*	72.99 ± 7.97*	49.63 ± 2.98*	16.73 ± 2.70	15.39 ± 3.05	46.17 ± 3.61
CpG-ODN (IT)	38.41 ± 3.11*	73.87 ± 6.70*	45.52 ± 2.03*	13.05 ± 0.97*	14.95 ± 2.92	40.97 ± 5.49
αPD1 + CpG-ODN (IT)	43.12 ± 3.46**	80.83 ± 7.62**	48.26 ± 3.53*	12.57 ± 2.17*	13.51 ± 1.43	42.45 ± 5.07
Treatment Non-Responders (TNR)	26.41 ± 1.90	49.39 ± 4.19	39.94 ± 2.01	21.05 ± 2.56	12.16 ± 2.69	43.03 ± 5.00

	CD4+ % of CD45+	CD4+ Teff % of CD4+	CD4+ Treg % of CD4+	CD19+ B % of CD45+	Eos cells % of CD45+	CD11c+ % of CD45+	m-MDSC % of CD45+
Control	12.88 ± 3.18	64.31 ± 11.70	12.38 ± 4.44	0.64 ± 0.10	0.35 ± 0.14	3.90 ± 0.54	2.26 ± 0.50
TR αPD1	15.99 ± 1.83	64.75 ± 7.97	12.65 ± 2.23	1.01 ± 0.28	0.43 ± 0.08	2.81 ± 0.77	5.46 ± 2.23
CpG-ODN (IP)	13.46 ± 1.63	69.75 ± 8.48	9.72 ± 5.16	0.97 ± 0.26	0.37 ± 0.18	4.25 ± 0.60	4.19 ±2.21
αPD1 + CpG-ODN (IP)	15.06 ± 3.61	62.27 ± 11.31	17.16 ± 6.01	0.62 ± 0.16	0.38 ± 0.06	3.52 ± 0.98	3.59 ± 1.53
CpG-ODN (IT)	40.97 ± 5.49	63.22 ± 14.48	15.16 ± 5.80	0.72 ± 0.22	0.34 ± 0.10	4.38 ± 0.68	5.71 ± 2.16
αPD1 + CpG-ODN (IT)	42.45 ± 5.07	60.98 ± 12.27	16.87 ± 7.17	0.51 ± 0.14	0.36 ± 0.13	3.21 ± 1.05	7.86 ± 5.30
Treatment Non-Responders (TNR)	43.03 ± 5.00	59.61 ± 9.45	12.83 ± 6.72	0.69 ± 0.29	0.39 ± 0.15	3.36 ± 0.88	4.63 ± 2.01

	CD4+ Naive % of CD4+	CD4+ Tcm % of CD4+	CD4+ Tem % of CD4+	CD8+ Naive % of CD8+	CD8+ Tcm % of CD8+	CD8+ Tem % of CD8+
Control	15.85 ± 2.89	4.37 ± 2.98	10.97 ± 7.06	4.51 ± 0.97	0.59 ± 0.43	9.22 ± 2.28
TR αPD1	17.94 ± 5.40	3.53 ± 1.90	9.60 ± 4.63	3.58 ± 1.51	0.35 ± 0.28	8.72 ± 2.01
CpG-ODN (IP)	12.00 ± 2.22	2.06 ± 1.06	11.56 ± 7.63	5.35 ± 2.61	0.81 ± 0.88	9.21 ± 1.70
αPD1 + CpG-ODN (IP)	17.71 ± 4.40	4.18 ± 2.29	11.63 ± 9.32	3.16 ± 1.38	0.31 ± 0.13	9.94 ± 2.96
CpG-ODN (IT)	11.43 ± 3.22	4.15 ± 1.39	15.75 ± 8.83	3.92 ±1.71	0.79 ± 0.46	13.98 ± 3.76
αPD1 + CpG-ODN (IT)	18.40 ± 10.30	4.67 ± 1.36	15.60 ± 3.61	2.06 ± 0.64	0.42 ± 0.16	12.16 ± 3.29

Treatment Non-Responders (TNR)	21.03 ± 10.79	4.15 ± 1.63	10.59 ± 5.41	2.66 ± 1.22	0.41 ± 014	12.50 ± 2.90
